# Androgens and spermatogenesis: lessons from transgenic mouse models

**DOI:** 10.1098/rstb.2009.0117

**Published:** 2010-05-27

**Authors:** Guido Verhoeven, Ariane Willems, Evi Denolet, Johannes V. Swinnen, Karel De Gendt

**Affiliations:** Department of Experimental Medicine, Laboratory for Experimental Medicine and Endocrinology, Katholieke Universiteit Leuven, Gasthuisberg, Herestraat 49, 3000 Leuven, Belgium

**Keywords:** testis, testosterone, androgen receptor, male infertility, microarray

## Abstract

Transgenic mouse models have contributed considerably to our understanding of the cellular and molecular mechanisms by which androgens control spermatogenesis. Cell-selective ablation of the androgen receptor (AR) in Sertoli cells (SC) results in a complete block in meiosis and unambiguously identifies the SC as the main cellular mediator of the effects of androgens on spermatogenesis. This conclusion is corroborated by similar knockouts in other potential testicular target cells. Mutations resulting in diminished expression of the AR or in alleles with increased length of the CAG repeat mimick specific human forms of disturbed fertility that are not accompanied by defects in male sexual development. Transcriptional profiling studies in mice with cell-selective and general knockouts of the AR, searching for androgen-regulated genes relevant to the control of spermatogenesis, have identified many candidate target genes. However, with the exception of *Rhox5*, the identified subsets of genes show little overlap. Genes related to tubular restructuring, cell junction dynamics, the cytoskeleton, solute transportation and vitamin A metabolism are prominently present. Further research will be needed to decide which of these genes are physiologically relevant and to identify genes that can be used as diagnostic tools or targets to modulate the effects of androgens in spermatogenesis.

## 1. Introduction

The testis is not only the main source of androgens, but it is also a key target for androgen action. Androgens play a vital role in the control of spermatogenesis. The mechanisms by which they exert this control, however, remain poorly understood. The unravelling of these mechanisms represents one of the major challenges in andrology. Here, we will present an overview of our present understanding of the cellular and molecular mechanisms by which androgens affect germ cell development with special emphasis on the contribution of a number of recently developed transgenic models in which androgen signalling in the testis is disturbed.

Spermatogenesis is controlled by a hierarchical network of regulatory systems. As nicely demonstrated by recent germ cell xenotransplantation experiments, germ cells themselves have a species-specific build-in programme that guides them through a unique and strictly timed sequence of steps of proliferation and differentiation (de França *et al*. 1998*a*; Brinster 2002). Successful completion of this programme, however, depends on a succession of signals and cues provided by the local environment (Skinner 1991; Verhoeven 1992; Jegou 1993; Mruk & Cheng 2004). This local network of regulatory factors is ultimately controlled by the endocrine system and in particular by the gonadotropins follicle-stimulating hormone (FSH) and luteinizing hormone (LH) that act as the master switches that turn the entire system on or off (Weinbauer & Nieschlag 1993; Sharpe 1994; McLachlan *et al*. 2002).

Somatic cells play a key role in the control of germ cell development. This is particularly true for the Sertoli cells (SC), epithelial cells that support developing germs cells structurally and functionally throughout their journey from the periphery to the centre of the seminiferous tubules where they are ultimately released as spermatozoa (Jegou 1993). At puberty and concordant with the onset of spermatogenesis, adjacent SC develop basally located specialized tight junctions that divide the tubules into a basal compartment containing mainly spermatogonia and an adluminal compartment containing spermatocytes and subsequent stages of germ cell development. In this way, SC not only shield developing germ cells from the immune system but also gain complete control of the specific humoral and nutritional environment, essential for germ cell development, in the adluminal compartment (Setchell & Waites 1975; Wong & Cheng 2005). SC communicate with adjacent germ cells by specialized cell junctions (gap junctions, ectoplasmic specializations (ES), tubulobulbar complexes), by environmental factors including nutrients such as transferrin and pyruvate, and by locally produced signalling molecules (Skinner 1991; Verhoeven 1992; Jegou 1993; Mruk & Cheng 2004). SC also affect Leydig cell development and function (Verhoeven 1992; Boujrad *et al*. 1995). Peritubular myoid cells play a key role in the production of the basal lamina surrounding the tubules and maintain bilateral interactions with SC and other testicular cells (Skinner 1991; Dym 1994; Verhoeven *et al*. 2000). They also contribute to the contractility of the tubules and the expulsion of spermatozoa. Leydig cells, apart from their role in androgen production, may provide other factors essential for germ cell development and maintain bilateral interactions with SC via cell signalling mechanisms that are only partially understood (Sharpe *et al*. 1990; Verhoeven 1992).

## 2. Androgens and the control of germ cell development

The main hormones controlling germ cell development are the gonadotropins FSH and LH. FSH acts via specific G-protein-coupled receptors which are widely accepted to be present exclusively on SC (Plant & Marshall 2001). One of its main actions is undoubtedly to promote SC proliferation in the immature testis (Orth 1984), but apart from this FSH exerts a variety of effects at different stages of germ cell development (Plant & Marshall 2001). LH acts on the Leydig cells and is responsible for the physiological levels of testosterone (T) in the male circulation and for the high intratesticular T levels (up to 100 times those observed in serum) that are apparently required to maintain normal spermatogenesis.

In a physiological setting, both FSH and androgens are required to initiate and maintain optimal spermatogenesis (Weinbauer & Nieschlag 1993; Sharpe 1994; McLachlan *et al*. 2002). Under a number of conditions, however, androgens are able to initiate, maintain or restore spermatogenesis in the (virtual) absence of FSH. Exogenous administration of physiological amounts of androgens—as used in most hormonal methods of male contraception—decreases LH and accordingly causes a drop in intratesticular T and a suppression of spermatogenesis. This effect can be overcome by pharmacological amounts of androgens, not only in rodents but also in higher species such as the monkey (for review, see Sharpe 1994). A key role for androgens in the control of spermatogenesis is further supported by a number of genetic models. In a mouse model of hypogonadotropic hypogonadism caused by a major deletion of the GnRH gene (*hpg* mice), androgens (but not FSH) are able to initiate qualitatively complete spermatogenesis including the production of fertile sperm (Singh *et al*. 1995; Handelsman *et al*. 1999; Allan *et al*. 2001). Along the same lines, fertility is maintained, be it at a reduced level, in mice (Dierich *et al*. 1998; Abel *et al*. 2000) and men (Tapanainen *et al*. 1997) with inactivating mutations of the FSH-receptor gene and in mice with a defect in the production of the FSH-β-chain (Kumar *et al*. 1997). Surprisingly, men with a deletion of the gene encoding FSH-β are azoospermic and infertile (Maroulis *et al*. 1977; Lindstedt *et al*. 1998).

An impressive amount of work has been done to define the specific contribution of FSH and androgens to the control of germ cell development in various species. As far as androgens are concerned, a variety of data indicate that, in the rat, stages VII and VIII of the spermatogenic cycle are particularly sensitive to androgen action. Withdrawal of intratesticular androgens by hypophysectomy (Russell & Clermont 1977), by chemical interference with gonadotropin production or action (Russell *et al*. 1981), or by administration of the Leydig-cell-specific cytotoxin ethane dimethanesulphonate (EDS) (Bartlett *et al*. 1986; Cameron *et al*. 1993; Kerr *et al*. 1993) results in a rapid increase in degenerating mid-pachytene spermatocytes and step 7 and 19 spermatids characteristic for stages VII and VIII. All these changes can be prevented or reversed by administration of LH or T (Russell & Clermont 1977; Kerr *et al*. 1993). Moreover, endogenous T concentrations have been shown to be higher at stage VIII than at any other stage of the cycle (Parvinen 1982) and immunohistochemical studies also reveal cyclic changes in the concentration of the androgen receptor (AR) in SC culminating at stage VII (Bremner *et al*. 1994; Vornberger *et al*. 1994). Parenthetically, a similar stage dependency of AR expression has also been observed in the human (Suarez-Quian *et al*. 1999). Finally, the most marked effects of T on tubular protein synthesis and secretion are also observed at stages VI–VIII (Sharpe *et al*. 1992).

At least four steps in germ cell development display clear-cut control by androgen action: spermatid adhesion and development, spermiation, progression through meiosis and spermatogonial differentiation. For the former three steps, there is an obvious relationship to stages VII and VIII of the cycle of spermatogenesis.

### (a) Spermatid adhesion and development

A widely used experimental paradigm to study the effects of androgens on spermatid adhesion and development are rats treated with silastic implants releasing low doses of T and oestradiol (TE implants). The main effect of these implants is that they suppress LH secretion and reduce testicular T levels to approximately 3 per cent of the control with no or only minor effects on FSH (McLachlan *et al*. 2002). As oestrogen receptors have been described in testicular somatic cells as well as in germ cells, the possibility of additional direct effects of the oestradiol on testicular cells may have to be considered (O'Donnell *et al*. 2001). One of the most striking effects observed after treatment with TE implants is a complete block in the conversion of round spermatids (RST) into elongated spermatids (EST) (O'Donnell *et al*. 1994). This block can be reversed by administration of high doses of androgens. Detailed stereological analysis reveals that conversion of RST between stages VII and VIII is the principal point of T action. The disappearance of RST and subsequent stages of germ cell development is accompanied by accumulation of large numbers of RST in the cauda epididymis, indicating that premature detachment of RST is an important underlying cause (O'Donnell *et al*. 1996). Attempts to elucidate the mechanism(s) by which T withdrawal results in premature sloughing of RST have largely focused on the potential role of apical ES, a testis-specific-actin-based type of adhesion junction that plays an important role in the adhesion of RST and EST to SC. In the rat, apical ES may already be noted at the pachytene phase of meiosis in stage VIII (de França *et al*. 1993). A marked increase in apical ES facing RST is noted in late stage VII and early stage VIII and they persist up to step 19 spermatids (O'Donnell *et al*. 2000). In the TE-treated rat, at a time when step 8 RST are detaching from SC, ES are apparently present and qualitatively normal, indicating that premature sloughing is not due to the absence of apical ES formation (O'Donnell *et al*. 2000). As a consequence the hypothesis has been advanced that detachment of RST may be due to a defect in the adhesion function of the relevant ES junctions. At least three protein complexes have been found to be associated with the apical ES and to be involved in their dynamic interactions with germ cells: the N-cadherin/β-catenin complex, the nectin/afadin complex and the α6β1-integrin/laminin-γ3 complex (Lee & Cheng 2004).

N-Cadherin has been suggested to be one of the mediators of androgen-dependent adhesion of spermatids. In the presence of FSH, T increases both the expression of N-cadherin and the adhesion of isolated spermatids to cultured immature rat SC (Cameron & Muffly 1991; Perryman *et al*. 1996). This adhesion is largely inhibited by an N-cadherin antiserum (Perryman *et al*. 1996). Surprisingly, similar effects of androgens were not observed when mature rather than immature SC were co-cultured with spermatids (Lampa *et al*. 1999). Moreover, in the mouse, oestradiol rather than T seems to be required for N-cadherin induction in the presence of FSH (MacCalman *et al*. 1997). Apart from the mentioned changes in N-cadherin expression, androgen withdrawal also results in a loss of N-cadherin/β-catenin association accompanied by a surge in Tyr phosphorylation of β-catenin. The exact mechanisms involved remain unknown, but a role has been suggested for c-Src and myotubularin-related protein 2. Interestingly, like N-cadherin, myotubularin-related protein 2 and c-Src are induced by androgens in SC-germ cell co-cultures and this induction is blocked by the antiandrogen cyproterone acetate (Zhang *et al*. 2005).

In a similar fashion, T-withdrawal-induced spermatid loss is accompanied by marked changes in the dynamics of the α6β1-integrin/laminin-γ3 complex. Under physiological conditions, adhesion between SC and spermatids is mediated at least in part by the α6β1-integrin/laminin-γ3 complex which, in turn, interacts with the activated focal adhesion kinase (pFAK)/c-Src complex in the SC to form cell-matrix focal adhesion-like structures. TE-induced spermatid loss causes dissociation of the pFAK/c-Src complex from the α6β1-integrin, activation of the extracellular signal-regulated kinase (ERK) signalling pathway and destabilization of the adhesion function of the apical ES (Wong *et al*. 2005).

### (b) Spermiation

Spermatid release or spermiation occurs at stages VII–VIII of the rat cycle and is very sensitive to T and/or FSH withdrawal (Russell 1993; McLachlan *et al*. 2002). Also in monkeys and men treated with T-based contraceptive regimens, spermiation failure followed by spermatid retention with subsequent phagocytosis by SC contributes considerably to the acute and chronic suppression of sperm counts. Spermiation failure can be induced both by selective withdrawal of androgens and by selective suppression of FSH, but marked synergistic effects between both hormones are evident (Saito *et al*. 2000; Matthiesson *et al*. 2006). The mechanisms by which hormone withdrawal impairs spermatid release remain poorly understood. Several aspects of the spermiation process, including the formation and degradation of tubulobulbar complexes and the removal of ES some 30 h before final spermatid disengagement, are apparently not affected by hormone withdrawal. Only the final disengagement between spermatids and SC seems to be impaired (Beardsley & O'Donnell 2003). Several lines of investigation suggest a role for α6β1-integrin and the associated phophorylated-FAK in the disengagement process, but the precise mechanisms involved and their hormonal control remain a matter of investigation (Beardsley *et al*. 2006).

### (c) Progression through meiosis

In a variety of experimental models, it has been demonstrated that suppression of endogenous androgen production or action in the testis results in a block early in meiosis and that concomitant administration of T or one of its active 5α-reduced metabolites permits completion of meiosis and formation of spermatids (Steinberger 1971). Experimental models used to support this contention include hypophysectomized rats treated or not with antisera directed against LH (Lostroh *et al*. 1963) or with antiandrogens (de França *et al*. 1998*b*) to further reduce endogenous androgen production or action, neonatal or adult rats receiving doses of oestrogens capable of suppressing gonadotropin secretion (Chowdhury & Steinberger 1975; Chemes *et al*. 1976) and immature rats treated with antisera directed against LH (Chemes *et al*. 1979). Studies in T-treated *hpg* mice (Handelsman *et al*. 1999) and recent transgenic models confirm the critical role of androgens in the control of meiosis (see §5*b*,*d*).

### (d) Spermatogonial proliferation and differentiation

It is widely accepted that FSH plays a major role in the control of spermatogonial proliferation (Sharpe 1994; McLachlan *et al*. 2002). Under some conditions, however, high intratesticular concentrations of T may interfere with these early stages of germ cell development. After irradiation, or administration of the toxic nematocide dibromochloropropane for instance, spermatogenesis in rats fails to recover owing to an inability of the early type A spermatogonia to survive and differentiate (Shuttlesworth *et al*. 2000; Meistrich *et al*. 2003). A similar defect is observed in a mouse strain that displays a spermatogenic arrest after the first wave of spermatogenesis (juvenile spermatogenic depletion or *jsd* mice; Shetty *et al*. 2006). In all these models, suppression of intratesticular T levels by administration of a GnRH analogue or by other means results in gradual recovery of spermatogonial differentiation, sometimes with complete recovery of spermatogenesis. This effect is enhanced by concordant administration of the antiandrogen flutamide, indicating the involvement of an AR-mediated process affecting testicular or extratesticular cells (Shetty *et al*. 2006). The nature of this unexpected effect of T remains elusive but studies in *jsd* mice suggest that T may indirectly affect testicular temperature and that temperature-dependent factors may be involved (Shetty *et al*. 2006). It is worth mentioning that high intratesticular T levels may also impede the development of transplanted spermatogonial stem cells. In fact, suppression of intratesticular T by pretreatment of recipient mice with a GnRH agonist markedly enhances colonization by donor cells (Ogawa *et al*. 1998).

## 3. Mechanisms of androgen action and control of spermatogenesis

The vast majority of the effects of androgens are mediated by the AR, a modular protein of approximately 110 kDa, encoded by a single gene located on the long arm of the X chromosome. The AR is a member of the large family of ligand-activated nuclear receptors. Inactivating mutations of the AR result in partial or complete forms of androgen insensitivity characterized by disturbances in the development of the normal male phenotype and disturbances in fertility (Quigley *et al*. 1995). For a more detailed discussion of the molecular mechanisms involved in androgen action, we refer to recent overviews (Gao *et al*. 2005; Heemers & Tindall 2007; Claessens *et al*. 2008). Here, we only present a schematic outline with special attention to elements that may be relevant to the mechanisms by which androgens control spermatogenesis.

The classical genomic pathway is generally considered the main pathway by which androgens exert their activities. In this pathway the AR is activated by interaction with natural or synthetic androgens. The activated receptor translocates to the nucleus, undergoes increased phosphorylation and binds as a dimer to recognition sequences known as androgen response elements (ARE), located in the regulatory regions of androgen responsive genes (Blok *et al*. 1996; Tyagi *et al*. 2000; Gao *et al*. 2005; Heemers & Tindall 2007). The structure of these AREs displays considerable variability. ‘Classical AREs’ are inverted partial repeats of 5′-TGTTCT-3′-like sequences separated by three nucleotides. They do not distinguish between the AR, the glucocorticoid receptor or the mineralocorticoid receptor. Apart from these classical AREs, however, ‘selective AREs’ have been identified that bind the AR more selectively. These selective AREs are organized as direct rather than inverted repeats of the 5′-TGTTCT-3′-like sequences and they display an alternative binding mode to the AR (Verrijdt *et al*. 2003). Binding of the AR to the DNA ultimately results in the recruitment of a wide variety of co-regulatory molecules or complexes which in turn induce changes in the conformation of the chromatin and/or facilitate interactions with the transcription initiation machinery (McKenna *et al*. 1999; Heemers & Tindall 2007). As a result, the expression of the targeted genes is modulated and specific proteins are induced or repressed. The mechanisms by which different genes are affected in distinct target cells are incompletely understood, but differences in chromatin structure, nature and number of available AREs, availability of the AR and specific co-regulators certainly play a role. It should be noted that not all the genomic effects of androgens are the result of direct interactions of the AR with AREs in the relevant gene. Many effects of androgens are indirect or secondary to androgen-induced changes in the production of transcription factors, paracrine mediators or hormones (Verhoeven & Swinnen 1999).

An important feature in the androgen signalling cascade is the role played by active metabolites. In many target tissues T, the main circulating androgen, is converted into 5α-dihydrotestosterone (DHT), a metabolite with a higher affinity for the AR and a higher intrinsic activity (Wilson 1975). Two 5α-reductases (type 1 and type 2) have been described that are responsible for the conversion of T into DHT (Russell & Wilson 1994). The vital role of DHT for some of the effects of androgens is illustrated by a typical form of pseudohermaphroditism (normal internal virilization, defective external virilization) observed in patients with congenital deficiency of the 5α-reductase type 2 (Wilson *et al*. 1993). Taking into account that the intratesticular concentration of T by far exceeds the concentration needed to saturate the AR, conversion of T into DHT may be less crucial in the testis except under conditions where the intratesticular concentration of T is decreased (McLachlan *et al*. 2002). Similarly, other effects of T depend on its conversion into 17β-oestradiol by the cytochrome P450 aromatase and subsequent activation of the cognate oestrogen receptors ERα and ERβ (Wilson 1975; Nilsson & Gustafsson 2002). Again, inability to catalyse this conversion or to activate the ER results in disturbances in some of the expected androgen effects (O'Donnell *et al*. 2001). The potential role of aromatization of T in the control of spermatogenesis will be discussed elsewhere in this issue.

Alternative signalling pathways have been suggested by which androgens may affect the function of target cells without a need for direct effects on gene expression (Silva *et al*. 2002; Walker 2003). These ‘non-genomic’ pathways may account for some effects of androgens that occur so rapidly that they cannot depend on AR-DNA-mediated gene activation. They could also provide a possible explanation for the intriguing observation that some effects of androgens in the testis seem to require concentrations of androgens that exceed the binding capacity of the classical AR. The nature of these signalling pathways and their potential role in the testis will be discussed separately in this issue.

When we try to apply this general knowledge on androgen signalling to the problem of androgens and the control of spermatogenesis, there are obviously a number of questions that need specific attention: (i) the target cell(s) by with androgens act to control germ cell development need to be identified; (ii) the molecular pathways that link androgen-mediated signalling to the control of germ cell development need to be unravelled; (iii) it remains to be explained why normal spermatogenesis apparently requires concentrations of androgens that clearly exceed those needed to saturate the classical AR; (iv) the role of active metabolites such as DHT and 17β-oestradiol in the control of spermatogenesis remains to be defined. In this overview, we will mainly focus on the first two questions.

## 4. Defects in androgen signalling and disturbed fertility in men

Deficient androgen production, whether owing to a primary defect at the level of the testis (Klinefelter syndrome, Leydig cell aplasia, genetic or drug-induced defects in androgen biosynthesis and so on) or to a dysfunction at the hypophyseo-hypothalamic level (Kallmann's syndrome, hypopituitarism, genetic or drug-induced defects in LH secretion and so on), is a well-known cause of male infertility. Disturbed androgen action, however, may also result in male infertility as clearly illustrated in patients with complete and partial androgen insensitivity syndromes (CAIS and PAIS). Moreover, there is growing evidence that more limited defects in androgen action may be responsible for some unexplained forms of male infertility (for overview, see Hiort & Holterhus 2003; Ochsenkuhn & de Kretser 2003).

More than 500 mutations of the AR resulting in CAIS or PAIS have been described to date. These patients present with variable degrees of feminization, maldescended testes and severely disrupted spermatogenesis not proceeding beyond the gonocyte or spermatogonial stages (Rutgers & Scully 1991). The aberrant location of the testes precludes unambiguous conclusions about the exact role of androgen insensitivity in the spermatogenic defect. Nonetheless, maturation of the seminiferous tubules and SC tends to be more advanced in patients with PAIS than in patients with CAIS (Muller 1984; Cassio *et al*. 1990). Moreover, even in patients with CAIS, there are sometimes indications for residual effects of androgens on the maturation of the seminiferous tubules. In fact, certain CAIS patients show some development of the epididymis or vasa deferentia, apparently reflecting residual activity of the AR in the presence of high concentrations of androgens during development. Interestingly, the same patients also display a higher degree of seminiferous tubule maturation at puberty than those without epididymides or vasa deferentia (Hannema *et al*. 2006).

The most convincing argument for impaired androgen action as a cause of idiopathic male infertility is the identification of a number of mutations of the AR resulting in patients that present with oligozoospermia or azoospermia despite an otherwise normal male phenotype (for overview, see Hiort & Holterhus 2003; Ochsenkuhn & de Kretser 2003). Some of these mutations are accompanied by a diminished capacity of the mutated AR to cause transactivation of reporter constructs *in vitro* or by a combined increase of T and LH *in vivo* reflecting impaired androgen responsiveness at the hypophyseo-hypothalamic level. The fact that only spermatogenesis is affected in these patients may indicate that this process requires a higher level of androgen responsiveness or that specific pathways of AR signalling are needed to sustain germ cell development. For example, in one patient, the mutated-AR allele impeded interaction of the AR with a specific co-activator, TIF2, known to play a role in androgen action in SC (Ghadessy *et al*. 1999).

Not only mutations but also polymorphisms of the AR may affect androgen responsiveness and fertility. The most extensively studied polymorphism is undoubtedly the length of the CAG repeat in exon 1, encoding a polyglutamine stretch in the aminoterminal domain of the AR. Extension of this repeat beyond its normal range (9–36 repeats) results in an X-linked recessive neurodegenerative disease (spinobulbar muscular atrophy or Kennedy's disease), characterized by progressive flaccid proximal paralysis and muscle atrophy as well as endocrine disturbances including gynaecomastia and progressive infertility (La Spada *et al*. 1991). Long CAG alleles have been associated with low intrinsic AR activity in reporter gene assays but also with the accumulation of toxic aggregates in androgen target cells. Interestingly, even within the normal range of variation of the CAG repeats, shorter repeat lengths seem to be associated with stronger androgen action and larger repeat lengths with weaker androgen action and disturbed spermatogenesis. An inverse correlation between repeat length and sperm concentration has been reported in normal men (von Eckardstein *et al*. 2001) and a large meta-analysis supports an association between increased CAG repeat length and idiopathic male infertility (Davis-Dao *et al*. 2007). However, these correlations could not be confirmed by all authors and in all populations studied, and the mechanisms involved remain a matter of debate. Apart from the decreased transactivation capacity of AR alleles with longer CAG repeats, and the accumulation of toxic complexes, increased CAG repeats seem to correlate with increased serum T levels (perhaps reflecting an attempt to compensate for the decreased androgen responsiveness) and increased oestradiol levels, which opens the possibility that some of the phenotypic consequences may be related to increased oestrogen receptor activation (Crabbe *et al*. 2007; Huhtaniemi *et al*. 2009). More complex relationships between CAG repeat lengths and male fertility have been described. For example, in hormonal contraception studies, longer repeats have been shown to increase the chances of becoming azoospermic in males with incomplete gonadotropin suppression (von Eckardstein *et al*. 2002). Similarly, the CAG repeat length has been shown to modify the susceptibility to adverse effects on semen quality linked to exposure to organohalogen pollutants (Bonde *et al*. 2008). The potential role of a GGN repeat, also located in the first exon of the AR, has been investigated less extensively, but short GGN repeats have been linked to decreased semen volume, possibly owing to suboptimal AR activity (Erenpreiss *et al*. 2008).

## 5. Conditional knockouts of the AR: identification of the SC as the principal mediator of the effects of androgens on the control of spermatogenesis

As in the human, a general knockout of the AR in mice results in a failure of the testes to descend in the scrotum, confounding studies on the role of androgen signalling in the control of spermatogenesis. The development of cell-selective knockout technology has created possibilities to circumvent this problem and has contributed considerably to our present understanding of the role of somatic cells and germ cells in the effects of androgens on spermatogenesis.

### (a) Candidate androgen target cells in the testis

Theoretically, androgens might act either directly on the germ cells or might affect germ cell development indirectly by acting on somatic testicular cells such as the SC and the peritubular myoid cells. A direct action on germ cells via the classical AR seems rather unlikely as the majority of evidence suggests that, at least in rodents, post-natal germ cells do not express the AR (Grootegoed *et al*. 1977; Bremner *et al*. 1994). Moreover, both chimeric mice carrying an AR-deficient cell line (Lyon *et al*. 1975) and mice receiving a testicular transplant of AR-defective spermatogonial stem cells (Johnston *et al*. 2001) are apparently able to produce fertile spermatozoa from the AR-deficient germ cells. These experiments indicate that the presence of a functional AR in somatic cells is sufficient to allow normal germ cell development. A possibility that cannot be ruled out is that androgens might affect germ cell development by a pathway not involving the AR.

If androgens act indirectly via somatic cells, SC are prime candidates given their intimate morphological and functional relationship with developing germ cells (Jegou 1993; Sharpe 2006). Adult SC unequivocally express the AR. Interestingly, however, AR expression in SC starts quite late (post-natal days 3–5 in rat and mice and from 4 years of age with a progressive increase to 8 years in men) and this could be the reason why they do not respond to the high intratesticular androgen concentration existing in foetal and early post-natal life (Bremner *et al*. 1994; Chemes *et al*. 2008; Willems *et al*. 2009). Once spermatogenesis has been initiated, cyclic changes in AR expression are observed both in rodents and in men suggesting that the germ cell complement affects AR concentration and androgen responsiveness in SC (Bremner *et al*. 1994; Suarez-Quian *et al*. 1999).

A third candidate androgen target cell is the peritubular myoid cell. These cells already express the AR during foetal development (Chemes *et al*. 1976; Merlet *et al*. 2007). Peritubular cells have relatively close contacts with spermatogonia; moreover, they display important bilateral interactions with SC that markedly affect both basal- and androgen-induced SC functioning (Skinner & Fritz 1985; Verhoeven & Cailleau 1988; Skinner 1991). At least part of these interactions may be mediated by one or more androgen-induced paracrine agonist(s) produced by peritubular cells and conveniently referred to as P-Mod-S (Skinner *et al*. 1988; Verhoeven *et al*. 2000). The nature of these agonist(s), however, remains enigmatic.

### (b) Selective ablation of the AR in SC

The development of conditional knockout technology has made it possible to explore more directly the role of the AR in putative testicular target cells. One of the first successful conditional knockouts in testicular cells was the SC-selective knockout of the AR (SCARKO) generated in our laboratory.

SCARKO mice were generated by *Cre*/*loxP* technology. In this technology, a key role is played by the Cre recombinase, an enzyme derived from the bacteriophage P1 that mediates efficient site-specific recombination between 34 bp recognition sequences known as *loxP* sites. If the relevant *loxP* sites are oriented in the same direction, recombination results in removal of the intervening (‘floxed’) DNA. If the *loxP* sites are oriented in opposite directions, recombination causes reversible inversion (Nagy 2000). To produce a cell-selective knockout of the AR, two strains of transgenic mice need to be crossed. The first strain should carry a mutated-AR allele in which a critical region of the AR is floxed. Importantly, this floxing should not disturb the production or activity of the AR in mice carrying the allele. The second mouse strain should express the Cre recombinase in a cell-selective way. Crossing of the two strains results in cell-selective removal of the critical region, and accordingly in inactivation of the AR.

**Figure 1.**
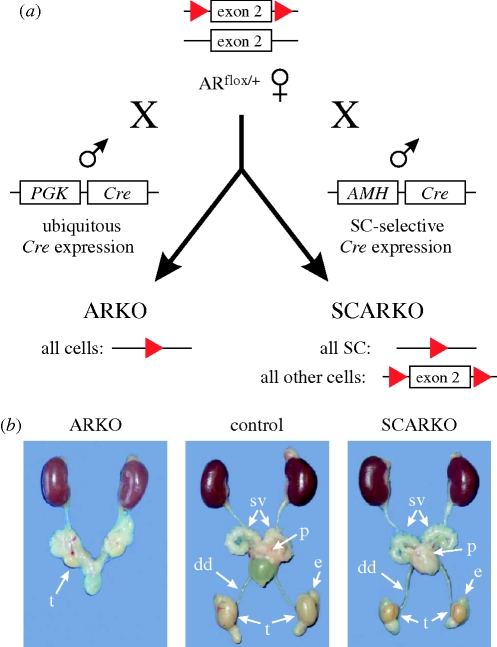
Generation and phenotype of mice with a general (ARKO) or Sertoli cell-selective (SCARKO) knockout of the androgen receptor. (*a*) Female mice, heterozygous for an AR allele with a floxed exon 2 (AR^flox/+^; *loxP* sites are indicated as red arrowheads) were crossed either with *PGK*-*Cre* mice, expressing the Cre recombinase ubiquitously, or with *AMH*-*Cre* mice, expressing *Cre* selectively in SC, to generate ARKO or SCARKO mice, respectively. (*b*) SCARKO mice have normally descended testes (t) that are markedly reduced in size (28% of control). Ductus deferens (dd), seminal vesicles (sv) and prostate (p) are normally developed. ARKO mice have very small testes that are located intraabdominally and male accessory sex tissues are absent.

For the generation of SCARKO mice, we developed a mouse strain in which exon 2 of the AR is floxed (AR^flox^). This exon encodes the first zinc finger of the DNA-binding domain of the AR which is crucial for the recognition of AREs (Quigley *et al*. 1995; Claessens *et al*. 2008). Moreover, removal of exon 2 disrupts the reading frame of the AR. The aminoterminal part of the AR can still be produced but is apparently instable and rapidly degraded (Hellwinkel *et al*. 1999). The second mouse strain expressing the Cre recombinase specifically in SC was developed by Dr F. Guillou (Tours, France; Lecureuil *et al*. 2002). This strain expresses the Cre recombinase under the control of a promoter derived from the anti-Müllerian hormone gene (*AMH*). Male mice carrying this *AMH*-*Cre* express the Cre recombinase selectively and uniformly in all SC and expression starts as early as day 15 post coitum. This implies that—in the SCARKO model—the AR gene is inactivated in SC more than a week before the onset of its expected physiological expression. SCARKO mice were produced by crossing females heterozygous for the mutant AR^flox^ allele with males expressing *AMH*-*Cre* (figure 1). As an additional control, mice with a general AR knockout (ARKO) were also generated by crossing AR^flox^ mice with *PGK*-*Cre* mice expressing the Cre recombinase ubiquitously under the control of the phosphoglycerate kinase promoter (Lallemand *et al*. 1998).

SCARKO mice display a unique and novel phenotype (De Gendt *et al*. 2004). In contrast with ARKO males or mice with a spontaneous general AR inactivation (*Tfm*), their external phenotype is male and their growth curve follows that of male littermates (table 1). Moreover, the typical male accessory sex organs (prostate, epididymis, ductus deferens, seminal vesicles) that are absent in ARKO and *Tfm* mice are normally developed in the SCARKO. Importantly, unlike ARKO and *Tfm* mice that display cryptorchidism, SCARKO mice have normally descended testes. However, despite their scrotal localization, these testes are reduced to 28 per cent of the size observed in normal adult littermates, suggesting severe impairment of spermatogenesis.

**Table 1.** Selected mouse models with defective androgen action. Quantitative values are expressed as a percentage of the control. Values that differ significantly (*p* < 0.05) are indicated by an asterisk. ND, not determined.

	ARKO	SCARKO	AR^flox(ex1-neo)/Y^	AR113Q	SPARKI
AR allele	deletion of exon 2	deletion of exon 2	floxed exon 1 neo-cassette in intron 1	CAG repeat length 113	substitution of exon 3 by GR exon 4
expression of mutant allele	ubiquitous	SC	ubiquitous	ubiquitous	ubiquitous
external phenotype	female	male	male	male	male
development of male reproductive tract	absent	normal	normal	normal	normal
testicular descent	defective	normal	normal	normal	normal
testis weight	8%*	28%*	81%*	∼50%	67%*
seminal vesicle weight	absent	108%	80%	ND	55%*
body weight curve	female	male	male	ND	male
serum LH	1435%*	178%	2429%	∼600%	113%
serum FSH	145%*	129%*	228%	∼23%	103%
spermatogenic defect	rare pachytenes	block in meiosis	late spermiogenesis	progressive defect	hypospermatogenesis

Immunohistochemistry confirms the complete absence of AR expression in the SC of SCARKO mice and the preservation of this staining in peritubular myoid cells and interstitial cells. Functional inactivation of the AR is confirmed by the loss of expression of *Rhox5*, a prototypic gene known to be directly regulated by the AR in SC. Interestingly, the number of SC (as measured by stereological evaluation of the nuclear volume) is unaffected in the SCARKO but drastically reduced in the ARKO (Tan *et al*. 2005). Despite the fact that some biochemical parameters (disappearing AMH expression and increasing p27^Kip1^, GATA-1, sulphated glycoprotein-2 expression) suggest normal maturation of SC, there are also signs of incomplete maturation (defective migration of SC nuclei to the periphery of the tubules; disturbed formation of SC tight barrier and so on; see §7*d*). Similarly, transcript levels of functionally important genes in SCARKO SC are variably affected (Tan *et al*. 2005; Denolet *et al*. 2006*a*). The functional deficiency of the SCARKO SC is most clearly illustrated by their inability to support normal spermatogenesis. In fact, the drastic reduction in testicular size is accompanied by a clear decrease in tubular diameter and in tubular lumen formation, observations that suggest decreased testicular fluid secretion, a process known to be controlled by androgens (Jegou *et al*. 1983; Au *et al*. 1986; Sharpe *et al*. 1994). Stereological analysis shows that ablation of the AR does not significantly affect the number of spermatogonia but reduces the number of spermatocytes, RST and EST to 64 per cent, 3 per cent and 0 per cent of the control, respectively, pointing to a block in meiosis (De Gendt *et al*. 2004). This block is confirmed by a clear reduction in the expression of several genes related to meiosis and the absence of expression of post-meiotic markers such as transition proteins and protamines (Verhoeven *et al*. 2007). Surprisingly, although basal as well as human chorionic gonadotropin (hCG)-stimulated levels of androgens in SCARKO mice are undistinguishable from those of normal controls (as also reflected by the normal weight of male accessory sex tissues) and although LH levels are not significantly increased, SCARKO mice display a clear disturbance in Leydig cell development (De Gendt *et al*. 2005). From day 20 onwards the number of Leydig cells is reduced by approximately 40 per cent. The remaining Leydig cells display an increase in size that probably contributes to the ultimately normal levels of androgens. The mechanism(s) by which AR ablation in SC affect(s) Leydig cell development remain(s) elusive. Known SC-derived growth factors such as platelet-derived growth factor alpha (PDGF-A) (decreased production in SCARKO) as well as unknown factors probably contribute (Tan *et al*. 2005).

Two other models with an SC-selective ablation of the AR have been described. The first one (the S-AR^−/y^ mouse) strongly resembles the SCARKO model (Chang *et al*. 2004). Here too, exon 2 is targeted for excision and SC-selective *Cre* expression is obtained using the same *AMH*-*Cre* strain described above. Only slight differences in phenotype are observed. The S-AR^−/y^ shows hypotestosteronaemia and a 4.5-fold increase in LH levels suggesting a more pronounced impairment of Leydig cell function. Nonetheless, seminal vesicle weight is not affected (Wang *et al*. 2006). Minor technical differences or diversity in genetic background might contribute to this slight variation in phenotype. In the second model, exon 1 is the targeted exon and *loxP* sites are oriented in opposite directions (Holdcraft & Braun 2004). An important feature of this model is that a hypomorphic phenotype is already observed in males carrying the floxed receptor, most probably as a result of the presence of a neomycin cassette, used in the generation of these animals, that was not removed. As a result, the floxed males (Ar^flox(ex1-neo)/Y^) have strongly reduced expression of the AR, a 19 per cent reduction in testicular weight and disturbances in the late stages of spermiogenesis near the time of spermiation (table 1). Epididymal sperm numbers are only 3.9 per cent of normal. LH and T levels are increased 23-fold and 40-fold, respectively. When females carrying this AR allele are bred to males of an *AMH*-*Cre* mouse strain (different from the one described above), inversion of exon 1 is observed with selective ablation of the AR in SC. Surprisingly, however, the reduction in testis weight is less pronounced than in the SCARKO or S-AR^−/y^ (60% of the control versus 28% and 29%), the block in spermatogenesis is observed at the level of the transition of RST into EST, and the reduction in Rhox5 expression is less pronounced than in the SCARKO. Taken together, these data suggest that in this model the knockout of the AR in SC is less complete, conceivably owing to the fact that *Cre*-mediated inversion is in principle reversible.

### (c) Selective ablation of the AR in germ cells, peritubular myoid or Leydig cells

Conditional knockouts ablating the AR in testicular cells other than SC have also been generated. A germ-cell-selective knockout (G-AR^−/y^) was produced using a mouse strain expressing *Cre* under the control of the synaptonemal complex protein 1 (*Sycp1*) gene promoter (Tsai *et al*. 2006). This promoter becomes activated at the leptotene–zygotene stage of meiotic development. No effects on spermatogenesis or fertility were observed. Complete AR ablation was seen in 15-week-old mice. Surprisingly, the efficiency of the ablation decreased rapidly, thereafter reaching 0 per cent at the age of 20 weeks. The mechanisms responsible for this decrease in AR inactivation remain elusive. Decreased *Cre* expression or methylation of *loxP* sites might be involved.

Mice with an ablation of the AR in peritubular myoid cells (PM-AR^−/y^) were generated using a mouse strain expressing *Cre* under the control of the transgelin (smooth muscle protein 22-α) promoter (*Tagln*-*Cre*) (Zhang *et al*. 2006). PM-AR^−/y^ mice displayed a 24 per cent reduction in testis size and oligozoospermia but fertility was preserved. Very likely, at least part of the observed defect was mediated by impaired SC functioning as the expression of many SC genes was affected. However, as *Tagln*-*Cre* mice were originally used to express *Cre* in vascular smooth muscle cells (Holtwick *et al*. 2002), a contribution of the latter cells to the testicular phenotype cannot completely be excluded.

Mice with an ablation of the AR in Leydig cells (L-AR^−/y^) were generated using a strain of mice expressing *Cre* under the control of the AMH type 2 receptor (*Amhr2*-*Cre*) (Xu *et al*. 2007). L-AR^−/y^ mice display a 67 per cent reduction in testicular size, a block in spermatogenesis at the RST stage in most testicular tubules and infertility. L-AR^−/y^ mice show disturbances in the expression of several steroidogenic genes and a marked reduction in T secretion, confirming the need for an active AR for normal Leydig cell development. The decrease in androgen production may largely account for the observed disturbances in spermatogenesis. As Amhr2 may also be expressed in SC (Jeyasuria *et al*. 2004), a contribution of the latter cells to the spermatogenic arrest, however, cannot completely be excluded.

### (d) A key role for the SC-AR

SC-selective knockout models such as the SCARKO show, for the first time unambiguously, that the SC is the main target cell by which androgens control spermatogenesis. In fact, the spermatogenic defect observed in these animals is as severe or even more pronounced than that observed in models in which androgen production in the testis is very drastically reduced, such as rats receiving TE implants or mice with an inactivating mutation of the LH receptor (Zhang *et al*. 2001; McLachlan *et al*. 2002; Lei *et al*. 2004). The central role of the SC is corroborated by studies on cell-selective knockout models targeting the AR in the other candidate target cells: peritubular myoid cells and germ cells. Furthermore, the SCARKO model proves that the classical AR is the main or only mediator by which androgens control progression through meiosis. Whether the AR acts exclusively through the genomic pathway or whether AR-mediated signalling through non-genomic pathways is also involved merits further investigation. Finally, the SCARKO model emphasizes that completion of meiosis critically depends on androgens. In this context, it is of interest to note that studies on the induction of spermatogenesis by androgens in *hpg* mice also suggest that meiosis represents the first and most sensitive step in the control of germ cell development by androgens (Singh *et al*. 1995; Handelsman *et al*. 1999). Additional conclusions are that the AR in SC is apparently not required for normal testicular descent, that it is also not needed to allow normal SC proliferation, but that normal Leydig cell development and function depend on an active AR in SC.

The SCARKO model does not exclude that some specific effects of androgens may be mediated by androgen action on cells different from SC. A nice example is the effect of androgens on SC proliferation. As already mentioned, animals with an ubiquitous KO of the AR have a reduced number of SC while the SC number is normal in the SCARKO, suggesting that this effect may be mediated by androgen action in other testicular cells such as the peritubular myoid cells (Johnston *et al*. 2004; Tan *et al*. 2005). The expression of a number of typical SC functional marker genes (including transferrin, N-cadherin) may also be modulated by androgen action in peritubular cells and this may help to explain the slight decrease in spermatogenesis observed in animals with a peritubular KO of the AR. Parenthetically, the above described inhibitory effects of androgens on spermatogonial differentiation observed in models such as the *jsd* mouse apparently also do not depend on androgen action in SC. In fact, recent experiments show that elimination of the AR in SC of *jsd* mice (by the generation of SCARKO-*jsd* mice) does not restore spermatogonial differentiation, whereas treatment of the same mice with a GnRH antagonist and flutamide stimulates recovery of spermatogenesis up to the spermatocyte stage (Wang *et al*. 2009). Replacement of flutamide by T reverses the stimulatory effect on spermatogonial differentiation, confirming that androgens are responsible for the observed arrest but also indicating that this effect is not mediated by the AR in SC.

An interesting extension of the SCARKO model has been the generation of mice with a double knockout of the FSH receptor (FSHRKO) and the AR in SC (SCARKO). These mice (FSHRKO–SCARKO) provide a unique baseline control from which the direct and specific actions and interactions of FSH and androgens on SC function and the control of spermatogenesis can be assessed (Abel *et al*. 2008). Comparison of germ cell development in the FSHRKO–SCARKO, the FSHRKO, the SCARKO and the WT control confirms that FSH specifically increases the number of SC and the number of spermatogonia. The initial onset of meiosis is apparently independent of direct hormonal regulation. In accordance with earlier data (de França *et al*. 1994), however, both FSH and androgens have marked effects on the maintenance of the meiotic germ cell population and at this level their effects seem additive. Completion of meiosis and entry in spermiogenesis are absolutely dependent on androgens. Spermiogenesis can apparently proceed without a requirement for FSH as the ratio of RST to mature sperm in the FSHRKO is similar to that in the control.

## 6. Other mouse models with mutant AR alleles affecting SC development and function

A few other mouse models have been developed that shed some light on the mechanisms mediating androgen action in the testis and on male forms of infertility related to disturbed androgen action (table 1).

As mentioned before, the Ar^flox(ex1-neo)/Y^ mouse represents a unique model of a partial defect in androgen sensitivity (Holdcraft & Braun 2004). This mouse carries an AR allele with a floxed exon 1 and a neomycin selection cassette in intron 1. It was actually produced to generate an SC-selective knockout of the AR by an approach slightly different from the one used for the SCARKO (see §5*b*). The presence of the neomycin cassette in the floxed allele, however, results in a hypomorphic phenotype in mice carrying this allele and causes a marked reduction in AR protein levels in different target tissues including the testis. By external examination, affected males are indistinguishable from WT littermates and they also show male sexual behaviour. T as well as LH levels are markedly increased, however, suggesting decreased AR function at the hypophyseo-hypothalamic level. Moreover, testis weight and epididymal sperm count are reduced by 19 per cent and 96 per cent of the control, respectively, and microscopic evaluation reveals marked disturbances in the later stages of spermatid differentiation near the time of spermiation. This model apparently strongly resembles the above mentioned minimal forms of androgen insensitivity in men and confirms that quantitatively normal spermatogenesis requires a higher level of AR function than male sexual differentiation. Moreover, it shows that a limited reduction in androgen responsiveness may disturb more selectively later stages of germ cell development.

A distinctive phenotype reflecting both partial and selective androgen insensitivity is observed in the SPARKI (SPecificity-affecting AR KnockIn) mouse (Schauwaers *et al*. 2007). In this model, the second zinc finger of the AR-DNA-binding domain has been replaced by that of the glucocorticoid receptor, resulting in a chimaeric AR that still binds classical AREs but is unable to recognize selective AREs. The SPARKI mouse displays normal male development and differentiation and normal levels of T and LH (unlike the Ar^flox(ex1-neo)/Y^), implying that selective AREs are not essential for male development and hypophyseo-hypothalamic feedback. Testis weight as well as prostate and seminal vesicle weight, however, are reduced to 67 per cent, 54 per cent and 55 per cent, respectively, suggesting a role for selective AREs in these target organs. The reduction in testis weight can be accounted for by a reduction of the number of SC (68% of control) but there also seems to be a decrease in the number of RST and EST per SC. SPARKI mice display normal mounting behaviour and remain fertile but both number and size of their litters are reduced by about 50 per cent. The involvement of selective AREs in the testicular phenotype is supported by the observation that on day 10, the testicular expression of *Rhox5*, a typical gene under the control of a selective ARE, is markedly reduced while the expression of two genes controlled by classical ARE (*Eppin*, *PCI*) is normal. The interesting feature of this model is that it confirms, in an *in vivo* system, that classical ARE and selective ARE drive distinct biological processes and accordingly that it may be possible to interfere with some of these processes (reproductive function) without affecting others (differentiation, behaviour, growth).

Two other transgenic mouse models have explored the effects of long CAG repeats on testicular function. In both models, very long repeat lengths were used. In the first model, a full length human AR carrying 120 CAG repeats was overexpressed in all tissues (McManamny *et al*. 2002). A progressive decrease in daily sperm production was noticed despite normal levels of T and LH, suggesting normal androgen action at the hypophyseo-hypothalamic level. The defect was suggested to be due to a toxic gain of function of the mutated protein rather than to a decrease in androgen responsiveness. In the second model, a 113 CAG repeat was inserted in the mouse AR (Yu *et al*. 2006). As in humans with Kennedy's disease, a progressive disturbance of germ cell maturation resulting in a decreased weight of the testis and infertility (from the age of 10 weeks on) was observed (table 1). Disturbances in the SC cytoskeleton were noted coinciding or preceding the defect in germ cell development. In contrast with the first model, normal T but increased LH levels were observed, suggesting diminished androgen responsiveness. *Rhox5* expression in the testis was normal, however, indicating that here too the disturbances in spermatogenesis are probably due to toxic effects related to the expanded repeat rather than to diminished androgen action.

## 7. Molecular mediators of androgen action in SC affecting germ cell development

The above summarized data clearly point to the SC as the main target of androgen action; still, the molecular mechanisms by which androgens ultimately affect spermatogenesis via the SC remain poorly understood.

### (a) Studies on isolated SC or SC lines

Studies searching for androgen-regulated genes in isolated SC or SC lines have yielded rather disappointing results. Disruption of the normal SC environment apparently causes a loss of expression of characteristic SC genes such as *Rhox5* and a marked decrease in androgen responsiveness (Denolet *et al*. 2006*b*). For a number of genes, androgen-induced upregulation (N-cadherin, cMyc, claudin 11, testin and so on) or downregulation (plasminogen activator, TGF-β1) can still be demonstrated, but the amplitude of the observed effects is limited (for review, see Verhoeven *et al*. 2008).

### (b) *Rhox5*, a lead gene in the search for molecular mediators of androgen action in SC

The most intriguing and strongly androgen-regulated gene identified in SC remains a homeobox gene originally named *Pem* and presently known as *Rhox5*. *Rhox5* was discovered by a screening for developmentally regulated genes and was subsequently found to be expressed in several reproductive tissues including testicular SC and principal cells of the caput epididymis (Lindsey & Wilkinson 1996). In the mouse testis, a massive androgen-dependent increase in expression is observed between day 8 and adulthood. Orthologues of *Rhox5* that are also controlled by androgens in the testis have been described in the rat and in the human (Sutton *et al*. 1998; Wayne *et al*. 2002).

In mice, *Rhox5* belongs to a large gene cluster of reproductive homeobox genes on the X chromosome (*Rhox*) devoted to regulate reproduction (MacLean *et al*. 2005). The gene has two promoters, a proximal promoter expressed in testis and epididymis and a distal promoter transcribed in extra-embryonic tissues and in ovarian granulosa cells (Shanker *et al*. 2008). The proximal promoter harbours four AREs (Hu *et al*. 2007) which selectively recognize the AR (Barbulescu *et al*. 2001). Targeted disruption of *Rhox5* results in increased germ cell apoptosis, a reduction in sperm count and motility and subfertility (MacLean *et al*. 2005). Several *Rhox5* target genes have recently been identified by microarray analysis. Marked effects have been observed on genes encoding proteins involved in metabolism, including the transcription factor PGC1α and the secreted proteins INS2, adiponectin and resistin. Unc5c, a netrin receptor known to trigger apoptosis in a ligand-dependent fashion, was shown to be negatively regulated by *Rhox5*. This finding is concordant with the fact that *Rhox5* ablation results in increased germ cell apoptosis (Hu *et al*. 2007).

### (c) Microarray studies on intact testes using ARKO models

As disruption of the normal cellular environment impedes gene expression and androgen responsiveness in isolated SC, identification of molecular targets of androgen action may only be possible in the intact testis. Three studies have used models with SC-selective or general ablations of the AR to search for such genes. Two additional studies have investigated the effects of exogenous androgens on hypogonadal or prepubertal mice testes.

**Figure 2.**
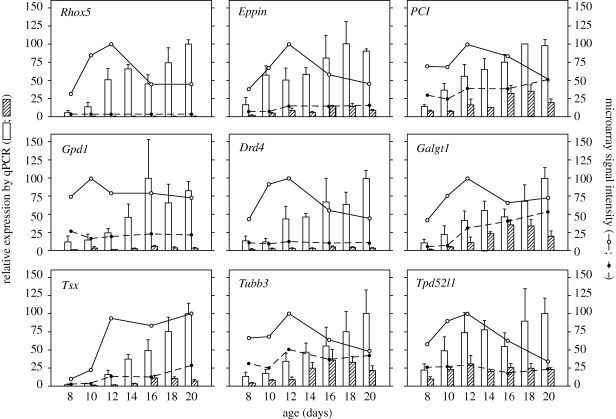
Expression pattern (from days 8 to 20) for a subset of genes originally identified as differentially expressed in SCARKO and control mice on day 10. The genes studied are *Rhox5* (*Pem*), *Eppin*, *Galgt1* (β-1,4-acetylgalactosaminyltransferase), *Drd4* (dopamine receptor D4), *Tsx* (testis-specific X-linked), *Gpd1* (glycerol-3-phosphate dehydrogenase 1), *Tubb3* (tubulin β3), *PCI* (protein C inhibitor) and *Tpd52l1* (tumour protein D52-like 1). Expression was assessed by microarray analysis and quantitative RT-PCR (qPCR). Left axis (bars): expression levels measured by qPCR in testes of control and SCARKO mice of the indicated ages (*n* = 3). Data were normalized to an external luciferase standard. All values are expressed as a percentage of the highest value measured for the corresponding gene arbitrarily set at 100. Values represent the mean ± s.e.m. of three measurements. Right axis (lines): gene expression measured by microarray analysis on a pool of mRNA from three testes of three control or SCARKO mice of the indicated ages. Data were expressed as a percentage of the highest signal observed for the studied gene, arbitrarily set at 100. Notice that qPCR confirms differential expression between SCARKO and control on day 10 for all the genes identified by microarray analysis. While the microarray data (reflecting the number of transcripts in a given amount of RNA) suggest a decrease in the transcript levels for most of the studied genes, and for some of them a loss of differential expression, the qPCR measurements (corrected for exogenously added luciferase and accordingly reflecting transcript levels per testis) show that this is an artefact caused by the increased contribution of developing germ cells to the total amount of RNA selectively in the control. The experiment illustrates that genes differentially expressed in SC may be missed by microarray analysis on samples with different degrees of germ cell maturation. Unfilled bar, control (qPCR); filled bar, SCARKO (qPCR); unfilled circle, control (microarray); filled circle, SCARKO (microarray).

A first AR-knockout study compared transcript levels in testes derived from 10-day-old SCARKO and control mice (Denolet *et al*. 2006*a*). Theoretically, SCARKO mice represent an ideal experimental paradigm to search for genes that depend on the AR in SC for their expression. The age of 10 days was selected as up to that time, at the onset of meiosis, the composition of the testis in SCARKO and control mice is indistinguishable and as effects of androgens on *Rhox5* expression were previously observed from day 9 on. Microarray analysis revealed that a surprisingly high number of genes (at least 692) were already differentially expressed on day 10. Forty of them (further referred to as ‘strongly regulated’) displayed a difference of at least twofold (28 downregulated and 12 upregulated in the SCARKO). The physiological relevance of the identified set of genes was supported by the observation that *Rhox5* displayed the highest degree of differential expression, and that the subset of strongly regulated genes included at least three genes (*Galgt1* (β-1,4-acetylgalactosaminyltransferase), *PCI* (protein C inhibitor) and *Eppin* (a serine protease inhibitor)) that were already known to cause male infertility when inactivated, and at least six genes (*Rhox5*, *Eppin*, *Gpd1* (glycerol-3-phosphate dehydrogenase 1), *Tubb3* (tubulin-β3), *Tpd52l1* (tumour protein D52-like 1) and *PCI*) for which there was previous evidence for androgen regulation in the testis or other tissues. Nine of the strongly regulated genes (*Rhox5*, *Eppin*, *Galgt1*, *Drd4* (dopamine receptor D4), *Tsx* (testis-specific X-linked gene), *Gpd1*, *Tubb3*, *PCI*, *Tpd52l1*) were studied in more detail. Decreased expression in the SCARKO from day 8 up to day 20 (figure 2) and preferential expression in the tubular compartment could be confirmed by quantitative RT-PCR (qPCR). Androgen regulation could also be confirmed in independent systems such as prepubertal mice treated with antiandrogens and organotypic cultures of 8-day-old testes (De Gendt *et al*. 2009). A functional analysis of the 692 differentially expressed genes pointed to an over-representation of genes involved in signal transduction, mitogen-activated protein kinase (MAPK) activity, cell adhesion, calcium binding, insulin-like growth factor (IGF) binding and, perhaps most strikingly, serine protease inhibition. In fact, five of these SERPINS were downregulated more than twofold in the SCARKO. A microarray time study (days 8–20) indicated that, apart from serine protease inhibitors, also serine proteases, cell adhesion molecules, cytoskeletal elements and extracellular matrix components displayed early and important differences in expression, supporting the hypothesis that tubular restructuring and changes in cell junction dynamics may be processes targeted by androgens during early puberty. As the AR in mouse SC appears from day 5 on, a complementary microarray study was performed on day 6 to search for genes that might display a very early (maybe transient) primary response to androgens and that might subsequently activate secondary cascades of androgen effects (Willems *et al*. 2009). Unfortunately, no such genes could be identified but the study confirmed that several of the genes identified in the day 10 study already display responses at or before day 6.

A similar microarray approach was applied to another SC-selective ARKO model, the Ar^flox(ex1-neo)/Y^; *AMH*-*Cre* mouse and to the floxed hypomorphic Ar^flox(ex1-neo)/Y^ mice used for the generation of this knockout (see §5*b*) (Eacker *et al*. 2007). As discussed, Ar^flox(ex1-neo)/Y^; *AMH*-*Cre* combine a ubiquitous form of partial androgen insensitivity with an SC-selective knockout of the AR that looks less complete than in the SCARKO. In this case, differential gene expression was studied at an age (8 weeks) where there are obviously differences in testicular cell composition between controls and mutants that may complicate interpretation of the data. Moreover, the markedly increased levels of LH observed in these AR mutants may be responsible for some of the observed effects on gene expression. The expression of 62 transcripts in the AR mutants (hypomorphs and selective knockouts) was found to differ by greater than twofold compared with the wild-type. Twelve transcripts were found to be uniquely affected in the SC-selective knockout and 16 additional transcripts were more affected in the selective knockout than in the hypomorph. Apart from *Rhox5*, none of the genes identified as putatively androgen-regulated, corresponded to the genes affected more than twofold in the 10-day-old SCARKO. Functional analysis suggested an over-representation of genes involved in metabolic processes and signal transduction. The cell adhesion molecule claudin 3 was also identified as a gene with a potentially important role (Meng *et al*. 2005).

A final study using ARKO models compared transcript levels in *Tfm* (ubiquitous knockout) and control mice on day 20. In total, 4700 differentially expressed genes were identified (O'Shaughnessy *et al*. 2007). Further *in silico* analysis and comparison with mice lacking germ cells identified 20 genes of a somatic tubular origin (including *Rhox5* and *Drd4*) that were significantly downregulated in *Tfm* testes and six that were upregulated. Genes downregulated in the *Tfm* testis were confirmed to be controlled by androgens by studies showing upregulation in T-treated *hpg* mice. Of the total number of genes differentially expressed in the *Tfm* testes, 50 per cent were associated with vitamin A metabolism, solute transportation or cytoskeletal function, leading the authors to suggest that androgens may affect spermatogenesis by modulating the tubular environment and by control of retinoic acid metabolism.

Two additional studies have used *hpg* mice or 8-day-old wild-type mice treated or not with T or DHT to search explicitly for early effects (4–24 h) of androgens (Sadate-Ngatchou *et al*. 2004; Zhou *et al*. 2005). These studies will not be discussed given the large differences in experimental design. For a more detailed discussion, see a previous overview (Verhoeven *et al*. 2008). It may be worth mentioning, however, that *Rhox5* was the only gene identified in common with the above discussed studies on ARKO mice.

It should be obvious from the data summarized above that there is very limited overlap between the sets of androgen-regulated genes identified in the available studies. This lack of consensus is undoubtedly largely owing to differences in experimental set-up.

Studies differ widely with respect to the age of the animals investigated, time and duration of androgen exposure, selectivity towards effects mediated by the AR in SC (as compared with the AR in other testicular cells), sensitivity to confounding factors such as secondary changes in gonadotropin secretion affecting testicular gene expression. Treatment/knockout-related differences in testicular cell composition also represent an underestimated confounding factor (figure 2). Accordingly, the data available should be interpreted with caution and should probably be regarded as complementary rather than contradictory.

### (d) Pathways involved in androgen-mediated control: topics for hypothesis-driven research

**Figure 3.**
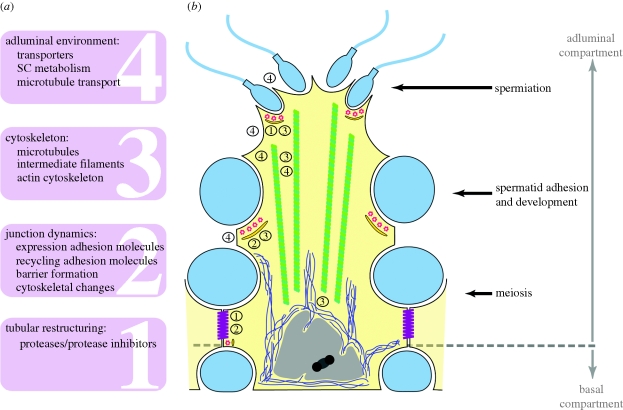
Molecular mediators of androgen action on spermatogenesis. (*b*) Indicates the main steps in spermatogenesis affected by androgens. In (*a*), a number of processes and molecular targets are summarized that are identified as potential targets of SC-mediated androgen action by the microarray experiments discussed in the text. The numbers in the centrally represented SC (yellow) refer to the processes summarized on (*a*). Developing germ cells are indicated in blue. Microtubules and intermediate filaments are represented in green and dark blue, respectively. Tight junctions are indicated in purple and the ectoplasmic specializations are represented by actin bundles (red) and a cistern of endoplasmic reticulum.

Despite the heterogeneity of the data available and the lack of consensus, some tentative conclusions seem appropriate:

— All the microarray studies, even those focusing on very early effects, identify relatively large sets of transcripts that are subject to androgen regulation. This suggests that androgens may affect the expression of a broad spectrum of genes rather than a few key genes. Further studies are obviously needed to confirm that all these putative target genes are indeed regulated by androgens, to find out which of them are expressed in SC, and to distinguish primary and secondary actions of androgens. In addition, however, and as illustrated in figure 2, it should be noted that microarrays comparing gene expression in testes with mutation/treatment-induced differences in cell composition may fail to identify several androgen-regulated genes expressed in SC.
— *Rhox5* remains the lead gene in the search for SC-specific genes directly affected by androgen action. It is the only gene invariably identified in all the summarized studies. Moreover, it is one of the very few androgen-regulated transcription factors identified. The finding that this gene is a member of a large homeobox family of genes, several of which also expressed in SC, may explain why, despite its suggested essential role, a knockout has only limited effects on fertility. Further investigations addressing androgen regulation and function of *Rhox5* orthologues in other species including the human are badly needed.
— Some functional families of transcripts seem to be prominently present in the lists of candidate androgen-regulated genes (figure 3). Proteases and protease inhibitors, cell adhesion molecules and cytoskeletal elements have been found to be over-represented in the microarray data (Denolet *et al*. 2006*a*; Eacker *et al*. 2007; O'Shaughnessy *et al*. 2007), suggesting that tubular restructuring (related to pubertal development and/or germ cell migration) and concomitant changes in cell junction dynamics may be important targets for androgen action. These changes together with modifications in cell metabolism and in the expression of several carriers involved in solute transportation (Eacker *et al*. 2007; O'Shaughnessy *et al*. 2007) point to a probable role of androgens in the creation of the specific and unique environment needed for germ cell development. Parenthetically, a more directed search for genes known to be important in SC function also revealed marked changes in the expression of transcripts encoding junction proteins (claudin 11, occludin, gelsolin) and cytoskeletal elements (vimentin) (Wang *et al*. 2006). Moreover, one of the most characteristic features of the tubular restructuring process, the formation of the SC barrier, has been shown to be disturbed in *Tfm* mice (Fritz *et al*. 1983) as well as in Ar^flox(ex1-neo)/Y^; *AMH*-*Cre* mice (Meng *et al*. 2005). This disturbance has been related to a decreased expression of the tight junction protein claudin 3 in the SC of the Ar^flox(ex1-neo)/Y^; *AMH*-*Cre* mice. Recent data indicate, however, that changes in two other tight-junction-related proteins, claudin 11 and Jam3, precede those observed in claudin 3 (Willems *et al*. 2009). Moreover, apart from their effects on gene expression, androgens may also affect junction dynamics by accelerating both endocytosis and recycling of junction proteins (including occludin, Jam-A and N-cadherin) via a clathrin-mediated pathway (Yan *et al*. 2008).
— An intriguing observation is that two studies identified several genes involved in vitamin A metabolism, suggesting a possible link between androgen action and retinoic acid metabolism and/or action (Eacker *et al*. 2007; O'Shaughnessy *et al*. 2007). Given the increasing amount of evidence that retinoic acid may play a role in the control of meiosis, the possibility of such a link certainly merits further investigation (Wolgemuth 2006). Interestingly, these genes were not identified in the SCARKO model and the mentioned studies were both based on animals with a ubiquitous (complete or incomplete) form of androgen insensitivity, suggesting that SC may not be the main or only target cells involved in these specific effects of androgens.

## 8. General conclusions

The development of transgenic mouse models displaying various defects in androgen signalling and the application of powerful transcription profiling techniques have contributed substantially to our understanding of the cellular and molecular mechanisms by which androgens control spermatogenesis.

A comparison of germ cell development in cell-selective knockouts of the AR in somatic cells (SC, peritubular myoid cells, Leydig cells) as well as germ cells of the testis unambiguously points to the SC as the main mediator of androgen action in the control of spermatogenesis. However, it is obvious that SC are very peculiar androgen target cells. The cyclic changes in SC-AR expression during the spermatogenic cycle and the finding that isolated SC display a loss in the expression of prototypic androgen target genes such as *Rhox5* and a loss of androgen responsiveness underline that the androgen responsiveness of SC depends critically on a specific microenvironment including the interactions of SC with germ cells. The mechanisms by which this environment controls the response of SC to androgens certainly merit further investigation. Androgen action on peritubular myoid cells may contribute to some specific effects such as the perinatal proliferation of SC and may modulate the expression of some SC genes, but these effects are apparently not crucial for male fertility.

The finding that selective and total ablation of the AR in SC causes a complete block in meiosis confirms the key role of androgens in the completion of meiotic progression and lends further support to the contention that meiosis may require lower concentrations of androgens than subsequent steps in germ cell development. Accordingly, the identification of androgen-regulated genes that play a decisive role in the control of meiosis is a central issue in our understanding of androgen action in spermatogenesis. Mice with a selective ablation of the AR in SC such as the SCARKO represent one of the most powerful tools to search for such genes. Whether the genes and pathways involved in androgen-mediated control of meiosis are the same as those involved in subsequent steps of spermatogenesis remains to be investigated. The complete block in meiosis developed by SCARKO animals implies that they may not be suitable to identify all the genes involved in post-meiotic effects of androgens. Generation of inducible knockout models that allow cell-specific as well as temporal control of AR inactivation may create novel experimental paradigms to overcome this possible limitation.

Selective defects in androgen action in SC have not been described in the human, but two recently developed mouse models, the hypomorphic mouse with diminished androgen expression (Ar^flox(ex1-neo)/Y^) and the mouse with an increased length of the CAG repeat of the AR, mimick human diseases in which spermatogenesis is specifically affected without obvious disturbances in male sexual development. A third model, the SPARKI mouse, suggests that it may be possible to interfere selectively with the effects of androgens on reproductive functions without impeding their anabolic actions on growth and skeletal development (table 1).

It is obvious that the search for molecular mediators bridging the gap between activation of the AR in SC and control of germ cell development has not yet resulted in definitive and unambiguous answers. The expectation or hope to identify one or a few key mediators of androgen action has not been fulfilled. In fact, transcriptional profiling studies in mice models with different defects in androgen signalling or in mice treated or not with androgens have invariably resulted in relatively large sets of genes that are putatively regulated by androgens. Whether key genes showing an early and direct response to androgens and initiating subsequent cascades of androgen effects exist is a question that still merits further investigation. The presently identified sets of androgen-regulated genes show surprisingly little overlap. As discussed above, this may largely be due to differences in experimental set-up or in the selected experimental paradigms. Moreover, despite the fact that many genes seem to respond to androgen action, the number of genes displaying a twofold or higher change in expression is limited, and for many genes confirmation of androgen regulation by independent techniques and in other experimental systems seems warranted. Furthermore, it is often unclear whether the genes identified are preferentially or exclusively expressed in SC and whether androgen regulation is direct or indirect. Despite all these limitations, a comparison of the lists of genes identified as being androgen-regulated with data from physiological studies on the steps of spermatogenesis that are most sensitive to androgen action suggests that a number of relevant functions and processes are prominently present (figure 3). Examples include genes involved in tubular restructuring, cell adhesion and junction dynamics, cytoskeletal organization, metabolism, solute transportation and vitamin A metabolism. The role of androgens in these processes is at present the subject of intensive research in several laboratories. It may reasonably be expected that these studies will soon result in reliable lists of genes reflecting androgen action in SC and in a better understanding of the molecular mechanisms by which androgens control spermatogenesis. A crucial question remains whether these genes, identified based upon mouse models, will also prove to be relevant mediators of androgen action in the testes of other species including men. Biochemical parameters of androgen action in human SC would be extremely valuable diagnostic tools to explore defects in androgen action in patients with idiopathic infertility. Moreover, some of these androgen-regulated genes might represent interesting targets to modulate the effects of androgens selectively in the testis and/or to develop novel male contraceptives.

## Note Added In Proof

While this review was in proof a paper appeared showing that—in contrast with previous observations (Zhang *et al.* 2006)—more appropriate inactivation of the AR in peritubular myoid cells causes major disturbances in spermatogenesis, comparable to those observed in SC-selective knockouts (Welsh *et al.* 2009). Also in this model, however, the effects on spermatogenesis may largely be mediated by secondary changes in SC function.
